# Phenotypic and genetic characterization of a novel phenotype in pigs characterized by juvenile hairlessness and age dependent emphysema

**DOI:** 10.1186/1471-2164-9-283

**Published:** 2008-06-12

**Authors:** Camilla S Bruun, Claus B Jørgensen, Lene Bay, Susanna Cirera, Henrik E Jensen, Páll S Leifsson, Jens Nielsen, Knud Christensen, Merete Fredholm

**Affiliations:** 1 Department of Basic Animal and Veterinary Sciences, Faculty of Life Sciences, University of Copenhagen, Grønnegårdsvej 3, DK-1870 Frederiksberg, Denmark; 2 Department of Veterinary Pathobiology, Faculty of Life Sciences, University of Copenhagen, Ridebanevej 3, DK-1870 Frederiksberg, Denmark; 3 National Veterinary Institute, Technical University of Denmark, Lindholm, DK-4771 Kalvehave, Denmark

## Abstract

**Background:**

A pig phenotype characterized by juvenile hairlessness, thin skin and age dependent lung emphysema has been discovered in a Danish pig herd. The trait shows autosomal co-dominant inheritance with all three genotypes distinguishable. Since the phenotype shows resemblance to the integrin β_6 _-/- knockout phenotype seen in mice, the two genes encoding the two subunits of integrin α_v_β_6_, i.e. *ITGB6 *and *ITGAV*, were considered candidate genes for this trait.

**Results:**

The mutated pig phenotype is characterized by hairlessness until puberty, thin skin with few hair follicles and absence of *musculi arrectores pili*, and at puberty or later localized areas of emphysema are seen in the lungs. Comparative mapping predicted that the porcine *ITGB6 *and*ITGAV *orthologs map to SSC15. In an experimental family (n = 113), showing segregation of the trait, the candidate region was confirmed by linkage analysis with four microsatellite markers. Mapping of the porcine *ITGB6 *and *ITGAV *in the IMpRH radiation hybrid panel confirmed the comparative mapping information. Sequencing of the *ITGB6 *and *ITGAV *coding sequences from affected and normal pigs revealed no evidence of a causative mutation, but alternative splicing of the *ITGB6 *pre-mRNA was detected. For both *ITGB6 *and *ITGAV *quantitative PCR revealed no significant difference in the expression levels in normal and affected animals. In a western blot, ITGB6 was detected in lung protein samples of all three genotypes. This result was supported by flow cytometric analyses which showed comparable reactions of kidney cells from affected and normal pigs with an integrin α_v_β_6 _monoclonal antibody. Also, immunohistochemical staining of lung tissue with an integrin β_6 _antibody showed immunoreaction in both normal and affected pigs.

**Conclusion:**

A phenotype resembling the integrin β_6 _-/- knockout phenotype seen in mice has been characterized in the pig. The candidate region on SSC15 has been confirmed by linkage analysis but molecular and functional analyses have excluded that the mutated phenotype is caused by structural mutations in or ablation of any of the two candidate genes.

## Background

A phenotype, characterized by juvenile hairlessness, thin skin and age dependent lung emphysema, has been encountered in a Danish pig herd. All piglets showing the trait are descendants of a specific boar and the trait shows autosomal co-dominant inheritance. At the age of six months hair growth is increased but not fully restored. A similar phenotype has been described in knock-out mice deficient of the *ITGB6 *gene (*ITGB6*-/-) encoding the integrin β_6_-subunit. These mice display juvenile hairlessness mainly on their neck, head and inner thighs, macrophage infiltration of the dermis and lymphocyte infiltration of the conductive airways of the lung. At the age of puberty, hair growth is resumed [1] and later, age dependent lung emphysema appears [2].

Integrins are a family of cell surface heterodimers, each consisting of an α- and a β-subunit. The integrin molecules are anchored in the cell membrane where they mediate a wide range of cell-cell and cell-matrix interactions [3]. The β_6_-subunit pairs only with the α_v_-subunit constituting integrin α_v_β_6 _[4]. The α_v_-subunit, however, pairs with five different β-subunits [5] and plays a central role with respect to normal embryonic development and survival. Ablation of *ITGAV *in mice causes malformations and embryonic or postnatal death [6].

The expression of α_v_β_6 _is restricted to epithelia and seems predominant in various developing organs of the embryo/fetus [7,8]. Besides, various patho-biological events induce a neo-expression of this integrin, e.g. tumor genesis [7,9], wound healing [10], and general cases of clinical or subclinical inflammation [7].

The resemblance between the phenotype detected in the pigs and the phenotype of the *ITGB6*-/- mice suggests *ITGB6 *as a candidate gene for the mutated pig phenotype. However, since the ITGB6 polypeptide dimerizes with ITGAV the gene encoding ITGAV is also considered a possible candidate.

In this paper the mutated pig phenotype is characterized macroscopically and histologically. Furthermore, taking the outset in linkage studies, sequencing and functional studies, *ITGAV *and *ITGB6 *are evaluated as candidates for the phenotype.

## Results

### Phenotype 0–6 months

**Figure 1:**
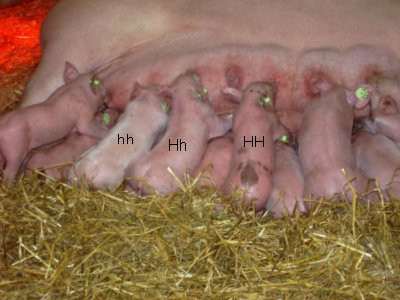
**Juvenile hairlessness in pigs**. All three genotypes (HH, Hh and hh) represented in one litter.

Based on the founder boar and his offspring an experimental population (n = 113) was established. A clear difference in amount of hair was observed in the pedigree, i.e. animals could be scored as normal (hh), heterozygous (Hh) and homozygous (HH) according to these observations. Hh pigs displayed hairless areas primarily on the back and rear, whereas HH pigs were almost entirely hairless. The degree of hairlessness varied somewhat within each genotype group. The skin appeared thin and reddish, mainly in the HH pigs but also in the Hh pigs (see Figure 1).

Shoulder and thigh skin samples from nine two months old pigs (three of each genotype) were evaluated histologically. The thickness of the dermis was measured and the hair follicles were counted. No lesions were observed apart from a few small areas with erosions and mild hyperkeratosis, which are normally present in the skin of piglets. However, in skin sections from both shoulder and thigh the dermis was significantly thinner in the HH and Hh groups compared to the hh group (p = 0.0001). There was no significant difference between the HH and Hh groups. The HH group had significantly fewer hairs/hair follicles than the Hh group (p = 0.0001), and the Hh group had significantly fewer hairs/hair follicles than the hh group (p = 0.01).

**Figure 2:**
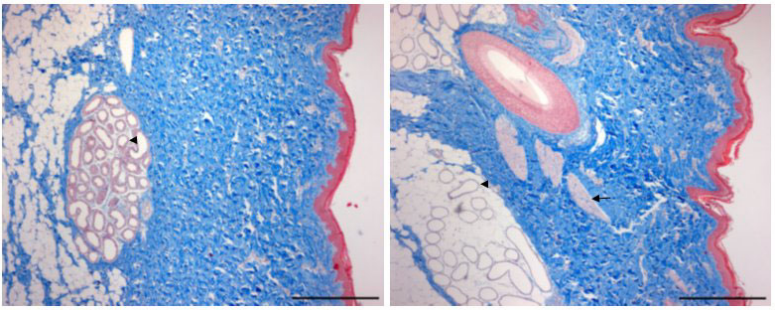
**HH and hh skin sections**. Skin from one HH (left) and one hh (right) pig, both two months old. The HH skin is characterized by a lack of hair/hairfollicles and *musculi arrectores pili *(arrow), thin dermis and sweat glands (arrowhead) embedded directly in the connective tissue (blue). Trichrome staining, bar = 200 μm.

*Musculi arrectores pili *were totally absent in skin from HH animals and only rarely detected in skin from Hh animals. Another morphologic difference was the tissue surrounding the sweat glands in the skin. In all skin sections these glands were found within the dermis close to the subcutis, but in HH skin they were embedded in connective tissue and in hh skin they were surrounded by fat tissue (see Figure 2).

Histological examination of lung tissue samples from the same nine pigs showed signs of interstitial pneumonia to a variable extend characterized by thickening of the alveolar walls due to septal infiltrates of mononuclear cells and hyperplasia of type II pneumocytes. Within some of the interstitial infiltrates syncytial cells were seen. Also hyperplasia of the bronchial associated lymphoid tissue (BALT) was present to a variable extend in all animals.

### Phenotype of adults

Around the age of six months, hair growth was resumed but even in adult HH pigs the hair coat was very thin and frizzy. Hh animals grew slightly more hair; however they did not recover normal hair growth. The skin of both HH and Hh adults became very scaly and histological examination of skin sections revealed noticeable hyperkeratotic epidermis. *Musculi arrectores pili *were still absent and the sweat glands were also surrounded by connective tissue in the skin of adult HH animals.

**Figure 3:**
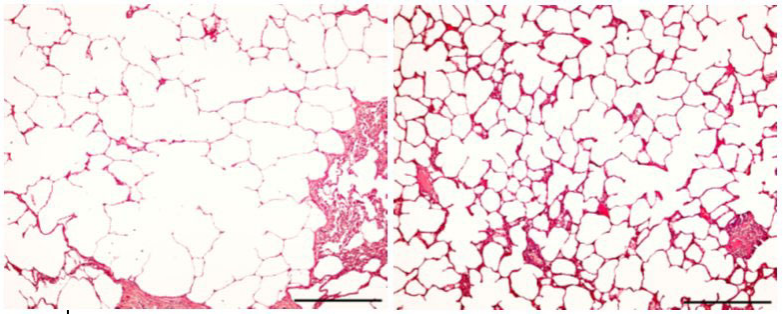
**Age dependent lung emphysema**. Lung from a HH boar of nine months, emphysema area (left) and normal area (right). Haematoxylin and eosin staining, bar = 200 μm.

Lung tissue of the HH animals (n = 5, ages from 8 to 21 months) and older Hh animals (n = 3, ages from 29 to 42 months) also showed interstitial pneumonia as well as areas with alveolar enlargement (localized areas of emphysema) (see Figure 3).

Lung tissues from one yonger adult Hh animal (n = 1, age of 12 months) and the hh animals (n = 4, ages of 8 months) showed normal, diffusely distended alveolar lumina. Some septal enlargement was observed in the lung tissue from the Hh and one hh.

### Candidate region

*ITGB6 *and *ITGAV *have been shown to be mapped to MMU2 at positions 34 and 46 cM respectively and to the orthologous region on HSAP2 at positions 160.7 and 187.2 Mb respectively [11,12]. Based on a human-porcine comparative map the *ITGB6 *and *ITGAV *candidate region was identified on SSC15 [13].

### Radiation hybrid (RH) mapping

The porcine *ITGB6 *and *ITGAV *genes were mapped by RH mapping. *ITGB6 *was mapped to the interval between the markers CL327053 and CL378417 [14] on SSC15 corresponding to an approximate position of 65 cM [13]. *ITGAV *was mapped to the interval between the markers CL356064 and CL360872 corresponding to an approximate position of 71 cM on SSC15 [13]. These results are in agreement with the orthologous positions of the human *ITGB6 *and *ITGAV*.

### Linkage study

**Figure 4:**
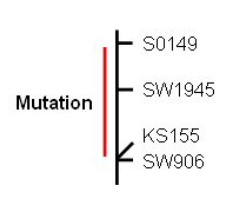
**Linkage region on SSC15**. The mutated locus has been mapped to a position between the markers S0149 and SW906 on SSC15.

A linkage study was performed with seven microsatellite markers selected from the candidate region on SSC15. The locus underlying the mutated phenotype was linked to four of the seven microsatellites: S0149, SW1945, KS155 and SW906 [15] with LOD scores from 8.4 to12.1. The mutated locus could be genetically mapped between markers S0149 and SW906 (~25 cM) with high confidence (LOD > 3), however finer mapping with SW1945 and KS155 could not be supported by a LOD score higher than 3. Keeping the linear order of the markers from the published linkage map [15] the result can be illustrated as shown in Figure 4.

### *ITGB6 *coding sequence

*ITGB6 *specific PCR amplification from skin cDNA samples gave either little or no PCR products, whereas the transcript was easily amplified in lung cDNA. PCR products covering the whole *ITGB6 *transcript were cloned and sequenced. The porcine *ITGB6 *coding sequence [GenBank: AM114538] consists of 2367 nucleotides covering 15 exons. A short 3'UTR of only 16 nucleotides precedes the polyA tail. The amino acid sequence [Swiss-Prot: Q1RPR6] has a similarity of 98.5 and 95.4% respectively to the human [Swiss-Prot: P18564] and mouse [Swiss-Prot: Q9Z0T9] ITGB6 amino acid sequences. It contains β6 specific features like the 2 Arg-Gly-Asp (RGD) sequences in the extracellular part and the 11 amino acid extension of the carboxyl terminus [16].

As observed for many other integrins [17] sequence analysis revealed alternative splice forms of the pre-mRNA. The following alternative splicing events were observed: skipping of exon 2 leading to frameshift and generation of a stop codon in exon 3; skipping of exon 6 (in frame); skipping of exon 12 through 15 with the concomitant inclusion of an additional sequence [GenBank:AM114539] that does not show homology to any of the splice variants reported in other species. In addition, there was evidence of concomitant skipping of exons 13 and 14. All splice forms of *ITGB6 *were detected in both hh and HH pigs. Alignment of the HH and hh *ITGB6 *coding sequences revealed one synonymous SNP but no allelic variants of this SNP showed cosegregation with the phenotype.

### *ITGAV *coding sequence

The porcine *ITGAV *coding sequence was generated by sequencing *ITGAV *specific PCR products amplified in lung cDNA. The porcine *ITGAV *coding sequence [GenBank: AM491473] consists of 3140 nucleotides and the 3' UTR consists of 831 nucleotides. The amino acid sequence [Swiss-Prot: A2RQD8] has a similarity of 95.8 and 94.2% respectively to the human [Swiss-Prot: A5YM53] and mouse [Swiss-Prot: A2AKI6] ITGAV amino acid sequences. No SNP's were detected in the alignment of the HH and hh *ITGAV *sequences.

### Quantitative PCR

Real time quantitative PCR (qPCR) was used to study expression of *ITGB6 *and *ITGAV *in animals with the hh and HH genotype respectively. In *ITGB6*, two different target sequences were included: one within exon 3 and one spanning exon 9 to 10. These exons seem to be present in all detected splice variants and the two target sequences provide an opportunity to compare the expression of two different parts of the transcript. In *ITGAV *the target sequence was spanning exon 1 to 3. The expression of *ITGB6 *and *ITGAV *was analyzed using REST [18] and the pair wise fixed reallocation randomisation test included in the programme. All three target sequences showed a slight upregulation in the HH group but the differences did not reach the significance level (p > 0.05) (see Table 1).

**Table 1:** *ITGB6 *and *ITGAV *qPCR

**ITGB6**		RPL4	YWHAZ	HPRT	Ex9-10	Ex3
	E	1.96	1.86	2.03	2.00	1.84
	SE	0.11	0.00	0.00	0.25	0.24
hh	Mean	18.31	20.37	22.99	26.17	27.16
	SE	0.08	0.24	0.17	0.24	0.21
HH	Mean	18.30	19.94	23.14	25.58	26.72
	SE	0.12	0.14	0.16	0.16	0.18
	Expression ratio				1.42	1.23
	p-value				0.073	0.078
**ITGAV**		RPL4	TBP	HPRT	Ex1-3	
	E	1.98	1.99	2.01	2.01	
	SE	0.14	0.00	0.00	0.00	
hh	Mean	25.11	21.91	24.01	21.54	
	SE	0.94	0.13	1.17	0.26	
HH	Mean	24.82	21.58	23.14	20.78	
	SE	1.04	0.28	0.51	0.28	
	Expression ratio				1.208	
	p-value				0.740	

qPCR data for the two *ITGB6 *target sequences Ex9-10 and Ex3 and the three reference genes *RPL4*, *YWHAZ *and *HPRT *and the *ITGAV *target sequence Ex1-3 and the three reference genes *RPL4*, *TBP *and *HPRT*. The hh group serves as control group for the HH group. All three p-values > 0.05 indicating not significantly different expression levels in the hh and HH groups.

### Western Blot

**Figure 5:**
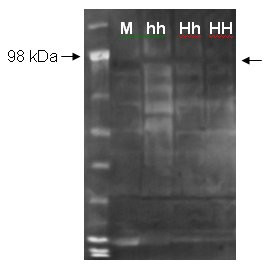
**ITGB6 western blot**. Western blot performed with lung protein from mouse (M) and three pigs representing the three genotypes (hh, Hh and HH). The left arrow points out the 98 kDa band of the marker. The right arrow indicates the ITGB6 specific bands of the pigs with approximate sizes of 98 kDa. The murine ITGB6 specific band has a comparable size of approximately 90 kDa.

Lung protein samples from one mouse (control) and three pigs representing the genotypes hh, Hh and HH were included in the western blot analysis. The detection was performed using an anti-mouse integrin β_6 _antibody. We observed a band of approximately 98 kDa in each of the pigs. These bands had an equal intensity and were comparable with the control mouse band of approximately 90 kDa (see Figure 5).

### Flow cytometry

**Figure 6:**
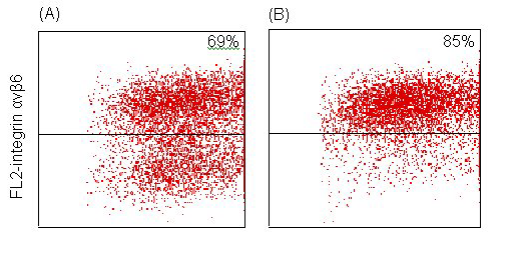
**Integrin αvβ6 flow cytometry**. SSC (sideward light scattering)/FL2 (fluorescence 2)-integrin avβ6 dot plots of primary kidney cells from 2 six-week-old pigs with genotypes hh (A) and HH (B), respectively. Dots in the upper right quadrant represent cells reactive with mAb 2077Z, and the percentage of integrin avβ6 positive cells is indicated for each pig.

The integrin α_v_β_6 _expression on kidney cells was compared between the hh (n = 2) and HH (n = 2) genotypes by flow cytometry. This experiment was included to study the expression in a non-lung tissue. For the two HH pigs, approximately 85% of the kidney cells were integrin α_v_β_6 _positive. For the hh pigs, the proportion of positive cells was lower, i.e. about 69%, as illustrated in Figure 6. The mean fluorescence intensity of positive cell populations, however, was at the same level for individual pigs (data not shown), showing a comparable integrin α_v_β_6_ expression of the two genotypes.

### Immunohistochemistry

**Figure 7:**
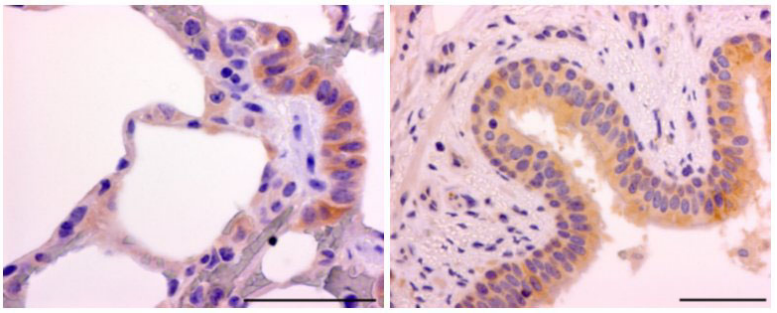
**Integrin αvβ6 immunohistochemical staining of lung tissues**. Lung tissues from all three genotypes stained with the integrin αvβ6 antibody. Representative pictures are shown. The red-brown colour localized to the alveolar, bronchiolar, bronchial and glandular epithelial cells indicates immunoreaction. Bar = 50 μm.

Immunohistochemistry was performed with goat anti-mouse integrin β6 antibody on lung tissue from seven pigs representing young and adult animals of the three genotypes (hh, Hh and HH). Lung tissues from all three genotypes stained with the antibody and the immunoreaction was localized to the alveolar, bronchiolar, bronchial and glandular epithelial cells (see Figure 7).

## Discussion

The mutated pig phenotype was originally detected in a boar and among his offspring. It is most probably caused by a spontaneous mutation in the founder boar and the corresponding phenotype has resemblance to that of the *ITGB6*-/- knockout mice. The only not coinciding features are the distribution of the hairlessness and the macrophage and lymphocyte infiltrations observed in the skin and lungs respectively of the knock-out mice. The interstitial pneumonia detected in the lungs of both young and adult pigs is most likely due to infection with porcine circovirus-2 (PCV-2) which is widespread within the Danish population of pigs [19] including our experimental population.

The fact that the hairlessness of the heterozygous pigs is intermediate indicates, that one functional copy of the gene in question is not enough to maintain a normal skin phenotype (or one dysfunctional copy can impair the "normal" pathway). The mutation seems to affect secondary structures of the skin in general. We have observed aplasia not only of hair follicles but also of *musculi arrectores pili*. Furthermore, the sweat glands are embedded directly in the connective tissue without any surrounding fat tissue. Also, the thickness of the dermis is affected.

Both heterozygous and homozygous pigs develop age dependent lung emphysema. Our animal material has not been sufficient to provide an "age of onset" of emphysema within the two genotype groups but our data suggest that emphysema appears around the age of puberty in homozygous animals and that the heterozygous pigs maintain a normal lung phenotype longer than the homozygous pigs.

Although the candidate region on SSC15 was confirmed, our results so far have not supported *ITGB6 *as a candidate gene. First, we did not detect any non-synonymous mutations in the coding region of the *ITGB6 *gene in the HH animals. A difference in the expression level of *ITGB6 *in the two genotype groups could have pointed towards a mutation in the regulatory region of this gene. Even if the *ITGB6 *expression was upregulated in the HH group, the difference was not significant. A reason for the upregulation could be the interstitial pneumonia observed in the HH group, as *ITGB6 *is known to be upregulated in response to inflammation [2,7,20]. In the hh group we observed interstitial pneumonia in only one pig. The western blot showed an apparently equal expression of the ITGB6 protein in the three groups.

The porcine gene encoding the integrin α_v _subunit (*ITGAV*) was mapped to a position on SSC15 within the candidate region. A mutation in *ITGAV *would be expected to have far more severe consequences, as the α_v _subunit pairs with five different β subunits [5]. In mice, for instance, the ablation of *ITGAV *causes embryonic or postnatal death [21]. Still, a mutation in *ITGAV *could not be entirely excluded as such a mutation might reside in a region interacting specifically with the β6 subunit. Sequencing of the *ITGAV *HH and hh coding sequences, however, did not reveal any potential causative mutation, and also, quantitative PCR results revealed an equal *ITGAV *expression in HH and hh pigs.

These observations were supported by the flow cytometric demonstration of high levels of the α_v_β_6 _receptor on kidney cells from HH animals and immunohistochemical detection of the receptor in HH lung sections. The presence of the receptor in HH pigs was furthermore confirmed by immunocytochemical staining of monolayer HH pig kidney cells (data not shown).

The pathogenesis underlying the hairlessness in the knockout mice is so far unknown. Huang *et al. *[1] observe fewer hair follicles and numerous degenerating hair follicles surrounded by mononuclear cells in the hairless dermis of the *ITGB6*-/- mice, indicating an inflammatory response. It has been demonstrated that the expression of *ITGB6 *is upregulated in response to injury and/or inflammation and that integrin α_v_β_6 _exerts a modulating effect on the inflammatory response [2,7,20]. Huang *et al*. [1] argue that the lack of integrin α_v_β_6 _in *ITGB6*-/- mice allows for minor skin irritations to cause an escalated inflammatory response resulting in hairlessness, for instance in the neck skin where the mother lifts her pups. Hair loss due to skin irritation can however, be excluded in the pig since the phenotype is inborn and without any sign of inflammatory response. Interestingly, our more detailed histological characterization of the skin show that hair follicles are present at a low frequency, however, without presence of *musculi arrectores pili*. This indicates that the mechanism underlying the phenotype has a subtle influence on cell migration/cellular development during embryonal development.

In contrast to the hairlessness the pathogenesis regarding the lung emphysema is well described in the *ITGB6*-/- mice. The condition is caused by an elevated expression of matrix metalloproteinase 12 (Mmp12), a macrophage expressed protease that degrades elastin causing a loss of lung elasticity and enlargement of alveolar airspaces. The elevated Mmp12 level, again, is caused by a lack of active transforming growth factor beta-1 (TGF-β1), which has a regulatory effect on the expression of *MMP12 *[2]. It has been shown that integrin α_v_β_6 _is central in activating TGF-β1. TGF-β1 is secreted in an inactive form bound to a latency associated peptide (LAP), but binding of α_v_β_6 _to the LAP part of the LAP-TGF-β1 complex, allows TGF-β1 to interact with its receptor [20]. Morris *et al. *[2] report a 200-fold increase in the *MMP12 *expression in *ITGB6 *knockout mice compared to wild-type mice.

Our results have supported neither *ITGB6 *nor *ITGAV *as candidate genes for juvenile hairlessness and age dependent emphysema in pigs. However, the resemblance with the *ITGB6*-/- knockout phenotype in mice is striking and it is obvious to expect the causative mutation in the pig to affect a pathway analogous to the murine integrin α_v_β_6 _– TGF-β1 – Mmp12 – pathway. This pathway is, however, complex and elaborately regulated [22,23] and involves several indispensable key elements in terms of maintaining full functionality. According to the database PigQTLdb [13] the region between S0149 and KS155 on pig chromosome 15 is syntenic to human chromosome 2 in the region from approximately 170 Mb to 220 Mb. According to Ensembl release 49, March 2008, this region comprises 350 protein coding genes many of which are poorly annotated. Thus, it will be necessary to narrow down the candidate region further, before good predictions of candidate genes possibly involved in the biochemical pathway leading to the phenotype in question can be made. It is, however, also important to keep in mind that we can not yet completely rule out that the integrin α_v_β_6 _is implicated in the phenotype since for instance a mutation in the promoter region or in a transcription factor/transcription factor target site could influence expression of either of the genes temporally or spatially.

## Conclusion

A mutated phenotype has been detected in the pig. The phenotype is characterized by hairlessness until puberty, thin skin with few hair follicles and absence of *musculi arrectores pili *and development of localized areas of emphysema in the lungs from the age of puberty. Linkage studies have unequivocally pointed out the region harbouring the mutation responsible for the phenotype. However, sequencing and functional studies of the most obvious candidate genes, *ITGB6 *and *ITGAV *have so far not supported either of them as the causative gene behind juvenile hairlessness and age dependent lung emphysema in pigs. Future work will therefore be directed at narrowing down the candidate region through linkage analysis in an extended experimental family. We are in the process of breeding additional animals for this purpose. Once the candidate region spans only a few megabases, re-sequencing will be performed in HH and hh animals with the purpose of identifying the mutation explaining the phenotype in question. Further characterization of the mutated gene or locus and the effect of the mutation will provide novel information about the biochemical pathway involved in the development of lung emphysema.

## Methods

### Experimental population and DNA

Based on the founder boar an experimental population of 113 pigs was established and an EDTA stabilized blood sample was collected from each animal. DNA was extracted using a salt precipitation method [24].

### Histopathology

From a two months old litter representing all three genotypes, three piglets of each phenotype were euthanized. Besides, 13 mature pigs (ages from eight to 42 months) were euthanized. Tissue samples from all lung lobes as well as skin samples from two locations on the lateral part of the left shoulder and thigh, respectively, were collected for histological evaluation. Following fixation for 24 hours in 10% neutral buffered formalin, the tissues were transformed to 70% ethanol for 48 hours. Tissues were processed through graded concentrations of ethanol and xylene, and embedded in paraffin blocks. The procedure of paraffin-embedment ensured that the 4–5 μm thick tissue sections, which were stained with haematoxylin and eosin or trichrome [25], were cut perpendicular to the tissue surface.

All tissue sections were examined histologically using an Olympus BX60 microscope. Besides, the thickness of the dermis and epidermis was measured (mm) (18 observations pr. animal) and the number of hairs (encompassing hair roots and hair follicles) was counted (4 counts pr. animal) from randomly obtained standard microphotos of each skin section. A Leica DC500 camera was used for this purpose and for all histological pictures presented. The data were analyzed using the generalized linear procedure (GLM), SAS^® ^System version 8.2 and a significance level of p < 0.05.

For immunohistochemistry, 3- to 4-μm sections of paraffin-embedded lung tissue from seven pigs representing young and adult animals of the three genotypes (hh, Hh and HH) were mounted on adhesive slides (Superfrost R Plus; Menzel-Glaser, Germany) and kept at 4°C until processed.

For the primary antibody (polyclonal goat anti-mouse integrin αvβ6 antibody) to work on porcine lung tissue, immunohistochemical stainings were initially performed with different procedures according to antigen retrieval methods and dilution of the primary antibody: a comparable positive reaction was obtained in sections of normal murine and normal porcine lung tissue when the concentration of primary antibody was 0.02 mg/ml and the antigen retrieval was performed with microwave oven boiling in Tris-EDTA buffer, pH 9 for 2 × 5 min. Before the antigen retrieval, the sections were heated at 70°C for 15 min. and then processed through xylene and rehydrated in graded concentrations of ethanol. After antigen retrieval and quenching of endogenous peroxidase activity (0.6% H_2_O_2 _for 15 min.) the sections were incubated with 5% normal rabbit serum (#X0902 DakoCytomation Norden A/S, Glostrup, Denmark) in TBS for 30 min. to reduce non-specific positive reaction. The sections were incubated with the primary antibody (#AF2389, R&D Systems Minneapolis, MN, USA) in 5% normal porcine serum in TBS, pH 7.6. for 18 h at 4°C, followed by washes in TBS. A secondary antibody (rabbit anti-goat #Z0454 DakoCytomation Norden A/S) was applied followed by wash in TBS. The sections were then incubated with goat peroxidase anti-peroxidase (#B0157 DakoCytomation Norden A/S) and positive reaction was visualized using the chromogenic substrate diaminobenzidine (#4170 Kem-En-Tec Diagnostics A/S, Taastrup, Denmark).

Counterstaining was performed for 10 s in Meyer's hematoxylin and washed 1 min. in running tap water and 4 min. in distilled water. Finally, sections were mounted with glycerol-gelatin. Control immunostainings were run on parallel sections without the primary antibody and with a nonsense polyclonal IgG antibody of the same concentration as the primary antibody. Sections of murine lung tissue served as positive controls for specific staining of integrin β6. The tissues were screened for reactivity following the optimal antigen retrieval procedure and dilution of the primary antibody.

### Linkage study

Seven microsatellite markers: SW118, S0149, SW1945, SW2608, SW1983, SW906 and KS155 [15] covering the region from 60 to 100 cM on SSC15 were selected and genotyped in the experimental family. Primers were labelled with fluorescent dyes and standard PCR were performed. Fragment analysis was done using an ABI (USA) PRISM^® ^3100 Genetic Analyzer and ABI GeneMapper™ Genotyping Software. For the linkage analysis the TWOPOINT, ALL and CHROMPIC options in CRI-MAP 2.4 [26] were used.

### *ITGB6 *coding sequence

Total RNA was isolated from lung tissue samples from two hh piglets using Qiagen (Germany) RNeasy Maxi kit according to the supplier's instructions. Four micrograms of the total RNA were incubated for 30 min at 37°C with RQ1 RNase-Free Dnase (Promega, USA) in a 20-μl reaction. The samples were then phenol extracted and precipitated. The DNAse treated total RNA was mixed with 2 μg of oligo dT_15 _primer and heated to 70°C for 5 min, placed on ice and reverse transcribed into cDNA using 400 U M-MLV Reverse Transcriptase and 50 U of Rnasin^® ^Ribonuclease Inhibitor (Promega). The reaction was incubated at 42°C for 1 h and terminated by heat inactivation at 95°C for 5 min.

For primer design the*ITGB6 *coding sequence from 5 species (Bos taurus (Genbank: NM_174698), Homo sapiens (Genbank: NM_000888), Mus musculus (Genbank: NM_021359) and Rattus norwegicus (Genbank: NM_001004263)) were aligned using "Multialin" [27]. Primers were designed from the consensus sequence in conserved regions that border a 1364 base pairs long central fragment of the *ITGB6 *coding sequence. The fragment was amplified using Elongase^® ^Enzyme Mix, Invitrogen™ (USA) and the primers ITGB6.2.F and ITGB6.2.R (see Table 2) according to the Elongase^® ^Enzyme Mix, Invitrogen™ protocol. The PCR products were run on a 1% agarose gel, appropriate bands were cut out and purified using GFX PCR DNA and Gel Purification Kit, Amersham Biosciences (UK).

**Table 2:** Primer sequences

Primer	Sequence
Exon15L	5'-CAGAGGCTCTACCAGCACCT-3'
Exon15_R2	5'-TTCGGGAGTAAAGCAGTTCT-3'
ITGB6.2.F	5'-CCTACATTTGGATTCAAGCAC-3'
ITGB6.2.R	5'-CTCCGTTTAGAGTTACAGGG-3'
GSP1	5'-CCCGCCAGTTTGCTGTCCATTCCAA-3'
GSP2	5'-GGGACTGTGTGTGCGGCAAGTGCAT-3
ITGB6_seq01L	5'-AGAACAAGTTCATCTGTATGAGAAT-3'
ITGB6_seq01R	5'-AAATGTTTCCATAAGGAGACAA-3'
Exon1+2_seqL	5'-TCTTTACCTGTCCAGGTAGC-3'
Ex2L	5'-ATGGGGATTGAACTGCTTTG-3'
Ex2R	5'-GGAGAGAGGGTTTTCGATGA-3'
Exon10_R	5'-TGCCTGCTTCTTTTCTCACA-3'
Exon9+10_seqL	5'-GTCTGAGGTGGAGCTGGAA-3'
Exon13L2	5'-CGAAGGGAAAACCATCATTC-3'
Exon3+4_seqL	5'-TTTACTTATCTCTCTGGAGTTGGAGA-3'
Exon9_11_L	5'-CCCACACCAAAAGAAATGCT-3'
MINF	5'-AAAGAGCTGAAGGAAGGTTTGA-3'
MINR	5'-GCTTTTTCACCGAAAGCAAG-3'
AV1F	5'-CGACAGGCTCACATTCTACTTG-3'
AV1R	5'-TCAGTCTCAGGGTTCTCCTTGT-3'
AV2F	5'-GGAACAGCTCTCAAAGTTTCCT-3'
AV2R	5'-GCACCTTCTCCTTGATTCTGAG-3'
AV3F	5'-AGGAGACTTCCAGACGACAAAG-3'
AV3R	5'-TCATGTTCTTGGAGTGACTTGG-3'
AV7F	5'-AGAGAGAGCCTGTTGGAACTTG-3'
AV7R	5'-CACTTCCCTTCAAGGATCTGAG-3'
AV8F	5'-GCTGTTTTAGCTGCTGTTGAGA-3'
AV8R	5'-TGTCCTCAATTGGAAGGTTCTT-3'
AV9F	5'-TCATTGAGGGAGATGTTCACAC-3'
AV9R	5'-ATGGGTCAGGATGTAGCGTAAC-3'
SGF	5'-ACTTCGGCGATGGCTTCT-3'
SGR	5'-CGGGTAGAAGACCAGTCACATT-3'
IN11F	5'-GATTTTCTGTCGCTGCCACT-3'
IN11R	5'-CAGCTTGGTCGTTTGGAAGT-3'
AVex1-2F	5'-ATG CAA CAG GCA ACA GAG ACT-3
AVex3R	5'-TCC TGA AGA AAG CAA GTT CCA-3'

The 5'- and 3'- *ITGB6 *specific sequence ends were amplified in the hh lung cDNA samples using BD SMART™ RACE cDNA Amplification Kit, BD Biosciences (USA) and the gene specific primers, GSP1 and GSP2 (see Table 2) following the user's manual. The PCR products were evaluated on a 1.2% agarose gel. The 5' and 3' PCR products showed distinct bands of approximately 1 and 1.4 kb respectively. These bands were purified using GFX PCR DNA Purification Kit, Amersham Biosciences.

For cloning of the PCR products Zero Blunt^®^TOPO^®^PCR Cloning Kit for Sequencing (Invitrogen) was used according to the manufacturer's instructions.

Plasmids were isolated from 6 colonies using QIAprep^®^Spin Miniprep Kit (Qiagen, Germany). Inserts were PCR amplified using T3 and T7 primers.

BigDye^®^Terminator v3.1 Cycle Sequencing Kit, Applied Biosystems (USA), was used for the sequencing reaction with T3, T7, M13, ITGB6.2.F and ITGB6.2.R primers according to the manufacturer's protocol. Sequencing was done using ABI PRISM^® ^3100 Genetic Analyzer. Based on the sequence information generated by analyzing these sequences, an additional set of sequencing primers, ITGB6_seq01L and ITGB6_seq01R (see Table 2) was designed for sequencing the central part of the *ITGB6 *coding sequence.

The generated trace files were base called and quality checked using PHRED [28], vector sequences were masked out using CROSS MATCH [29], repetitive sequences were masked using REPEATMASKER [30], sequences were assembled into contigs using PHRAP [29] and viewed using CONSED [31].

### Sequencing of *ITGB6 *coding sequence in HH

cDNA from two HH animals was generated as described for the two hh animals. The *ITGB6 *was PCR amplified using the primers Exon1+2_seqL and Exon15_R2 (see Table 2) and Phusion™High-Fidelity DNA Polymerase (Finnzymes, Finland) according to the manufacturer's instructions. The PCR products were sequenced with these primers as well as Ex2L, Ex2R, Ex6R, Exon10R, Exon9+10_seqL and Exon13L2 (see Table 2) as previously described.

The HH sequences were assembled with the hh sequences as described earlier and the assembly was scrutinized for sequence differences between the two genotype groups.

### *ITGAV *coding sequence

Seventeen porcine EST clones [32] with inserts homologous to the human *ITGAV *coding sequence [Genbank: NM_002210] were cultured overnight at 37°C on LB agar plates [33] containing 0.01% carbaniciline. One colony representing each clone were inoculated in LB broth [33,33] containing 0.01% carbaniciline. Plasmids were isolated using QIAprep^®^Spin Miniprep Kit (Qiagen) according to the manufacturer's instructions. The purified plasmids were cut with the restriction enzymes *Xho*I and *Eco*RI at 37°C overnight and inserts were PCR amplified using T3/T7 or T3/2390R. PCR products were sequenced using BigDye^®^Terminator v3.1 Cycle Sequencing Kit (Applied Biosystems) and the same primers. Based on consensus sequences from the clones and the human *ITGAV *coding sequence, eight primer sets (AV primers, see Table 2) were designed to cover almost the entire porcine *ITGAV *coding sequence. Two porcine shotgun sequences (BL_75693 and BDE_82477) [34] aligning with the human *ITGAV *coding sequence were used to design the primer set (SG primers, see Table 2) for amplifying the 5' end of the coding sequence. The nine *ITGAV *primer sets were used in standard PCR reactions in lung cDNAs from one hh and one HH pig. PCR products were sequenced using BigDye^®^Terminator v3.1 Cycle Sequencing Kit, (Applied Biosystems).

HH and hh sequences were assembled as described for the *ITGB6 *sequences and the assembly was scrutinized for sequence differences between the two genotype groups.

### RH mapping of *ITGB6 *and *ITGAV*

The porcine *ITGB6 *and *ITGAV *were mapped in the IMpRH panel [14,35,36]. For *ITGB6 *a standard PCR reaction was applied with the primers: Exon15L (annealing in exon 15) and Exon15_R2 (annealing in the 3'UTR, see Table 2). For mapping of *ITGAV*, intron 11 was first PCR amplified and sequenced as described previously using the primers IN11F and IN11R (see Table 2) annealing in exon 11 and 12 respectively. RH mapping was performed with the primers MINF and MINR (both annealing in intron 11, see Table 2). PCR amplification in the 118 hybrids was evaluated after electrophoresis on a 1.2% agarose gel.

### Quantitative PCR

Lung RNA samples from each of four HH pigs (from eight to 22 months old) and four hh pigs from another farm (eight months old) were generated using TRI REAGENT^®^, Molecular Research Center (Ohio) according to the manufacturer's instructions. RNA samples were DNAse treated using RNeasy^® ^Mini Kit (Qiagen). cDNAs were synthesized as described earlier.

For *ITGB6 *the cDNA was diluted five fold. Two primer sets were used: one within exon 3 (Exon3+4_seqL and Ex2R, see Table 2) and one spanning the exon 9/exon 10 boundary (Exon9_11_L and Exon10_R, see Table 2).*Ribosomal protein L4 *(*RPL4*), *hypoxanthine phosphoribosyl-transferase *(*HPRT*) and *tyrosine 3-monooxygenase/tryptophan 5-monooxygenase activation protein, zeta-polypeptide *(*YWHAZ*) were included in the study as reference genes.

For *ITGAV *the cDNA was undiluted and the primers AVex2F (annealing at the exon1-exon2 boundary) and AVex3R (annealing in exon 3) (see Table 2) were used. The reference genes were *Ribosomal protein L4 *(*RPL4*), *hypoxanthine phosphoribosyl-transferase *(*HPRT*) and *TATA binding protein *(*TBP*).

An MX3000p™ thermocycler (Stratagene, USA) was used for the real time quantitative PCR. For each primer set a standard curve was constructed using a dilution series of known concentrations of purified PCR products (QIAquick^® ^PCR Purification Kit (250), Qiagen). The standard curves represented a plot of CP (crossing point, defined as the cycle at which the fluorescence signal crosses the threshold) vs. the relative concentration of PCR product. Single PCR reactions were performed for each cDNA sample, a genomic DNA sample and a non-template negative control using Brilliant^® ^SYBR^® ^Green Master Mix (Stratagene). SYBR green fluorescence was automatically detected during each PCR reaction. The MX3000p™ software (Stratagene) was used for baseline adjustment and accordingly for setting the fluorescence threshold value. CP values for the HH group obtained from all three target sequences was compared to CP values for the hh "control" group using REST [18] with 2000 randomisations. A significance level of p < 0.05 was chosen.

### Western Blot

Protein was isolated from lung tissue samples from mouse, one hh pig, one Hh pig and one HH pig using TRI REAGENT^®^, Molecular Research Center (Ohio) following the manufacturers instructions. Eighty μg of each protein sample as well as ten μl of the marker SeeBlue^®^Plus2 (Invitrogen) were loaded on a NuPAGE™ 4–12% Bis-Tris gel and run for 55 minutes at 200 V using the XCell *SureLock*™ Mini-Cell, Invitrogen. The proteins were blotted onto a PVDF membrane, Invitrogen, using an XCell II™ Blot Module, Invitrogen, according to the manufacturer's protocol. The membrane was stained with Ponceau S, Sigma (USA) to verify successful transfer of proteins. Detection was performed using WesternBreeze^® ^Chemilunimescent Western Blot Immunodetection Kit, Invitrogen and the antibody Anti-mouse Integrin β6 Antibody (#AF2389, R&D Systems Minneapolis, MN, USA). A concentration of 0.4 μg antibody per ml primary antibody diluent was used. A BioMax Light film, Kodak, was exposed for 10 minutes.

### Flow cytometry analysis

An indirect three-step immuno-fluorescence staining method for porcine B-cells [37] was adapted to label trypsinised primary monolayer kidney cells from the 4 pigs (2 HH and 2 hh). A volume of 100 μl cell suspension (10 × 10^6 ^cells/ml) was incubated with 25 μl (10 μg/ml) of the anti- integrin α_v_β_6 _mAb MAB2077Z (Chemicon International Inc, Denmark). After washing with 2 ml PBS supplemented with 0.1% sodium azide (washing buffer), the cells were incubated for 30 min with 25 μl (1:100 dilution) biotin-conjugated goat-anti-mouse (BIOGAM) IgG (Jackson Immuno Research Laboratories (JIRL) Inc., West Grove, PA, USA) diluted in washing buffer. After another washing, the cells were incubated with 25 μl (1:50 dilution) R-phycoerythrin (RPE)-conjugated streptavidin (R0438, DAKO, Denmark) for 10 min. After washing, the cells were finally resuspended in 200 μl BD FACSFlow™ (Becton Dickinson) for flow cytometric examination. All incubations were performed at room temperature in the dark and centrifugations were carried out at 490 × *g *at 24°C. Controls without any reagents and with secondary antibody, only, were included for each pig. In each test, 10,000 cells were counted using a FACScan flow cytometer (Becton Dickinson) for data acquisition, and data were analysed using the Cell-Quest™ software (Becton Dickinson).

## Authors' contributions

CSB carried out the main part of the genetics laboratory work, the sequence and linkage analysis, tissue sampling and drafted the paper. CBJ assisted in the sequence and linkage analysis. LB carried out the *ITGAV *sequencing, RH mapping and qPCR. SC participated in the RNA extraction, qPCR analysis and the western blotting. HEJ and PSL carried out the histological and immunohistochemical studies. JN carried out the flow cytometry analysis. KC established the experimental population and carried out the SAS analysis. MF initiated the study, carried out the comparative mapping, blood and tissue sampling and coordinated the work. All authors contributed to and approved the final manuscript.
